# Alterations in cell growth and signaling in ErbB3 binding protein-1 (Ebp1) deficient mice

**DOI:** 10.1186/1471-2121-9-69

**Published:** 2008-12-18

**Authors:** Yuexing Zhang, Yan Lu, Hua Zhou, Myounghee Lee, Zhenqiu Liu, Bret A Hassel, Anne W Hamburger

**Affiliations:** 1 Department of Pathology, University of Maryland School of Medicine, Baltimore, Maryland, USA; 2 Greenebaum Cancer Center, University of Maryland School of Medicine, Baltimore, Maryland, USA; 3 Department of Oral and Maxillofacial Surgery, Shanghai Jiaotong University School of Medicine, Shanghai, PR China; 4 Department of Epidemiology, University of Maryland School of Medicine, Baltimore, Maryland, USA; 5 Department of Microbiology and Immunology, University of Maryland School of Medicine, Baltimore, Maryland, USA

## Abstract

**Background:**

The ErbB3 binding protein-1 (Ebp1) belongs to a family of DNA/RNA binding proteins implicated in cell growth, apoptosis and differentiation. However, the physiological role of Ebp1 in the whole organism is not known. Therefore, we generated *Ebp1*-deficient mice carrying a gene trap insertion in intron 2 of the *Ebp1 (pa2g4) *gene.

**Results:**

Ebp1^-/- ^mice were on average 30% smaller than wild type and heterozygous sex matched littermates. Growth retardation was apparent from Day 10 until Day 30. IGF-1 production and IGBP-3 and 4 protein levels were reduced in both embryo fibroblasts and adult knock-out mice. The proliferation of fibroblasts derived from Day 12.5 knock out embryos was also decreased as compared to that of wild type cells. Microarray expression analysis revealed changes in genes important in cell growth including members of the MAPK signal transduction pathway. In addition, the expression or activation of proliferation related genes such as AKT and the androgen receptor, previously demonstrated to be affected by Ebp1 expression *in vitro*, was altered in adult tissues.

**Conclusion:**

These results indicate that Ebp1 can affect growth in an animal model, but that the expression of proliferation related genes is cell and context specific. The Ebp1^-/- ^mouse line represents a new *in vivo *model to investigate Ebp1 function in the whole organism.

## Background

Members of the ErbB receptor tyrosine kinase family (ErbB1-4) and their ligands are important regulators of cell growth and differentiation. Studies of ErbB1, ErbB2 and heregulin (the ErbB3/4 ligand) deficient mice indicate that these genes are essential for embryonic development [1]. In turn, the activity of the ErbB receptors is regulated by their interacting partners. An ErbB3 binding protein (Ebp1) was cloned in our laboratory during a yeast two-hybrid screen [2]. Ebp1 is identical to the murine p38-2G4 protein which was isolated as a DNA binding protein[3]. These proteins are members of the Proliferation-associated 2G4 *(Pa2g4) *gene family, which is highly conserved throughout evolution [4]. More than 30 genes encoding proteins homologous to Ebp1 have been found in organisms ranging from *Danio rerio *to *Pan troglodytes *[5].

Ebp1 is expressed in mammalian cell lines derived from multiple origins. Ebp1 mRNA is also found in all normal adult human and murine tissues examined including liver, heart, brain, placenta, lung, muscle, pancreas, kidney, prostate and breast [6,7]. Examination of EST data bases reveals that Ebp1 is expressed in all tissue types at different stages of embryonic development. Its ubiquitous distribution in both embryonic and adult tissues suggests it affects various developmental and physiological pathways [6].

An important role for Ebp1 in cell proliferation and survival *in vitro *has been demonstrated by many groups. We have shown that Ebp1 inhibits transcription of E2F1 regulated cell cycle genes such as E2F1, Cyclin D1 and cyclin E [8]. This transcriptional regulation is due in part to interactions with the transcriptional corepressors Sin3A, Rb and HDAC2 on E2F1 regulated promoters [9,10]. Ectopic expression of *Ebp1 *inhibits the growth of human breast[11] and prostate cancer cells [9,12] and fibroblasts [13] both *in vitro *and in animal models [14]. In breast cancer cell lines, Ebp1 regulates levels of ErbB2 and controls the cellular response to heregulin and the antiestrogen tamoxifen [15]. In prostate cancer, ectopic expression of Ebp1 results in downregulation of Androgen Receptor (AR) and several of its target genes and inhibition of AR-regulated cell growth [14]. Ebp1 also has a role in regulating cell survival as its interaction with AKT kinase suppresses apoptosis[16].

The mechanisms by which Ebp1 exerts its effects on cell proliferation and survival are incompletely understood. The biological effects of Ebp1 were originally postulated to be based on its ability to bind DNA. Ebp1 is a member of the SF00553 protein superfamily, the prototype of which is a 42 KDa DNA binding protein isolated from the fission yeast *S. pombe *[17]. Blast analysis reveals that Ebp1 and the yeast 42 KDa protein have 38% amino acid identity and 56% similarity. The *S. pombe *protein preferentially binds a synthetic curved DNA sequence. Ebp1 binds directly to this synthetic curved DNA sequence and is recruited to E2F1 promoter elements *in vitro *and *in vivo *as part of a protein complex [18]. The interaction of Ebp1 with the E2F1 promoter is regulated by the ErbB3 ligand HRG.

In addition to its ability to interact with proteins and DNA, Ebp1 binds to an array of RNA targets. Squaritto et al [13] found that a pool of EBP1 localized to the nucleolus binds RNA and may be involved in ribosomal biogenesis. EBP1 can bind to B23 (nucleophosmin), as a component of pre- ribosomal ribonucleoprotein complexes [19]. In the cytoplasm, Ebp1 associates with the 40 S subunit of mature ribosomes, suggesting that it can also be involved in protein translation. Sedimentation studies revealed that Ebp1 copurified with eIF2α, a component of the translation initiation complex [20]. Ebp1 contains an RNA binding domain σ- 70 like motif that maps to aa 46–64 that mediates its interaction with RNA and its nucleolar localization [13]. A double-stranded RNA binding domain was also mapped to aa 91–156. Other studies showing that EBP1 binds the 3'UTR of bcl-2 mRNA and stabilizes β-globin-ARE bcl-2 transcripts [21] suggest that EBP1 is a functional RNA binding protein. Finally, Ebp1 has been demonstrated to bind to viral internal ribosome entry sites (IRES) [22].

Despite burgeoning interest in the role of Ebp1 in several important cellular activities, the physiological function of Ebp1 in the intact organism is not known. We therefore characterized Ebp1- deficient mice generated by a gene trap insertion in intron 2 of the *pa2g4 *gene. We show that Ebp1-deficient mice exhibit transient dwarfism. This is accompanied by a decrease in serum levels of IGF-1 and IGF binding proteins and changes in expression of several other genes associated with cell growth. These results provide *in vivo *evidence that Ebp1 plays an important role in cellular growth.

## Results

### Disruption of the Ebp1 gene

**Figure 1:**
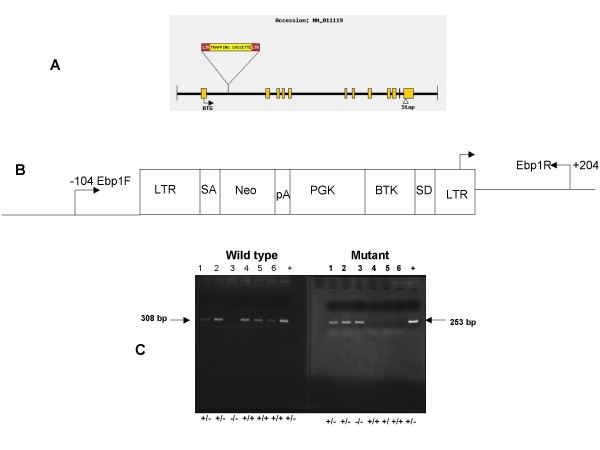
**Generation of the Ebp1 null mouse**. A. Schematic representation of the retroviral integration site within the first intron of the mouse *pa2g4 *(*Ebp1*) gene in the ES clone OST 186047 is indicated. Exons are represented as boxes. B. The locations of the PCR primers (LTR2, Ebp1 R, Ebp1 F) used in genotyping are indicated. Their positions (in base pairs) relative to the sites of integration are shown (not drawn to scale). LTR = long terminal repeat; SA and SD = splice acceptor and splice donor sites, respectively; PGK = phosphoglycerate kinase 1. C. A typical genotyping analysis showing the 308 bp PCR fragment for a wild type allele and the 253 bp product that detects the viral integration site. The products were separated by agarose gel electrophoresis and visualized by ethidium bromide staining.

Ebp1^+/- ^mice were generated at Lexicon Genetics from ES cells containing a retroviral gene trap in the *Ebp1 *locus as described previously [23]. Sequence analysis showed that the gene trap was inserted at a single site in the 2.3 kb intron 2 of the *Ebp1 *gene (Fig. 1A, B). The insertion led to the expression of an Ebp1-neomycin fusion molecule containing only the first 20 amino acids that are encoded by Exon 1 of *Ebp1 *that has no known biological function. The mice were genotyped by PCR with primers located at each side of the gene trap and in the LTR2 cassette (Fig. 1C).

**Figure 2:**
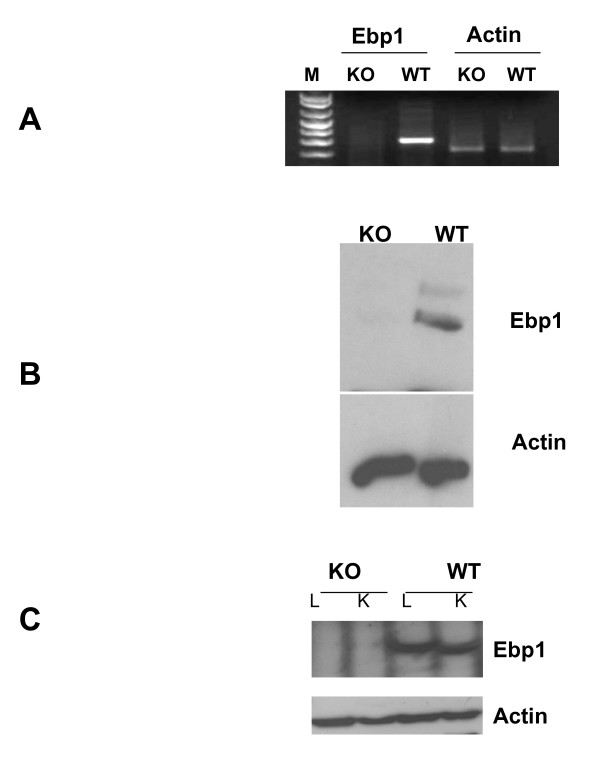
**Ebp1 is not expressed in Ebp1^-/- ^animals**. A, B. Fibroblasts were derived from 12.5 day Ebp1^-/- ^(KO) and wild type (WT) embryos as described in the Materials and Methods. A. RNA was isolated and the expression of Ebp1 and actin was determined by conventional PCR analysis. B. The expression of Ebp1 in KO and WT MEFs was analyzed by Western blot analysis. C. The expression of Ebp1 from lung (L) and kidneys (K) of 10 week old Ebp1^-/- ^and wild type littermates was detected by Western blot analysis.

The absence of *Ebp1 *mRNA in *Ebp1*^-/- ^mice was confirmed by RT-PCR with β actin as an internal control on total mRNA from 12.5 day embryo fibroblasts. The transcript was present in fibroblasts from wild type, but not Ebp1^-/- ^MEF (Fig. 2A). In addition, Western blot analysis indicated that Ebp1 protein was not expressed in knock out MEFs (Fig. 2B).

### Ebp1 expression in adult life

Examination of EST data bases and our own published data [6] indicate that Ebp1 is ubiquitously expressed in normal adult mouse tissue. We therefore measured Ebp1 protein expression in several organs to determine the penetrance of the knock out. We found as expected that Ebp1 was expressed in adult lung and kidney of 10 week old wild type mice. Ebp1 expression was absent in the Ebp1^-/- ^mouse (Fig. 2C).

### Breeding data and post natal lethality

Breeding data were obtained from crosses between both heterozygous and homozygous mice, since both Ebp1^+/- ^and Ebp1^-/-^male and females were fertile. In all cases, the sex ratio was approximately 1:1. The genotypes of the pups from heterozygous pairs were present at approximately the expected ratio of 25:50:25 (Ebp1^+/+^, Ebp1^+/- ^and Ebp1^-/-^) (n = 40). Survival was approximately equal for all three groups eight weeks after birth. Male and female knock-out mice were fertile and inbred mating resulted in viable offspring. However, litter size was smaller for the Ebp1^-/- ^mice. Litter size of wild type mice was 9.1 ± .8, and for knock out mice,4.2 ± 1.3 (4 litters for each group).

In general, life expectancy, of knock out mice was indistinguishable compared with their littermates. Ten mice of each genotype were maintained for one year. 9/10 knock out and 10/10 heterozygous and 10/10 wild type mice survived.

### Reduced growth of Ebp1^-/- ^mice

**Figure 3:**
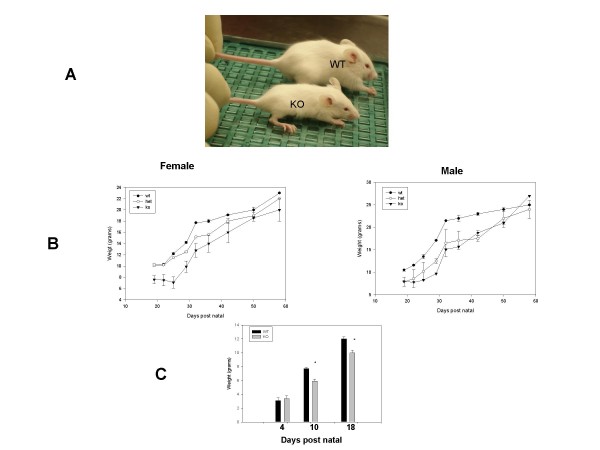
**Growth retardation of Ebp1^-/- ^mice**. A. Photograph of wild type and Ebp1^-/- ^male littermates at Day 30. B. Wild type, Ebp1^+/- ^and Ebp1^-/- ^littermates, derived from crosses of heterozygous parents, were weighed over a period of 60 days. Results are plotted as the mean body weight ± standard error of the mean. C. Wild type and Ebp1^-/- ^male and female mice, the progeny of crosses of homozygous animals, were weighed at the days indicated. Two litters each were weighed KO = 9 mice, WT = 15. (*p = .013 at Day 10, p = .02 at day 18).

The most striking feature of adult *Ebp1*^-/- ^mice is their initial smaller body size (Fig. 3A) and in about 1/3 of the mice the presence of kinky tails. The progeny of crosses between heterozygous parents were kept with the heterozygous mother and weighed sequentially. At day 19, there was a significant (p = .008) reduction of approximately 30% in body weight for both males and females. Mice were weaned at Day 21. After Day 30 mice began to catch up in size with wild type litter mates and at Day 60 there were no differences in weight (Fig. 3B). To rule out the possibility that competition during milk sucking was responsible for growth retardation, Ebp1^-/- ^males and females or wild type males and females were crossed. KO or WT progeny were housed alone with their mother. Males and females were weighed and data pooled due to difficulties in determining sex at early time points. Weights were the same for WT and KO mice at Day 4. However, at Days 10 and 18, these mice also displayed a 25% reduction in weight (2 litters, 9 total knock out, 15 total wild type).

### Macro and microscopic appearance of organs

To identify development defects and or pathological features, we examined the macro and microscopic appearance of a series of organs from adult Ebp1 mice. There were no gross morphological differences between the *Ebp1*^+/+ ^and *Ebp1*^-/- ^mice at 10 weeks of age. The examination of hematoxylin and eosin stained sections from organs revealed no histological changes in gut, kidney, lymph nodes, testes, liver, spleen, pancreas, skeletal muscle, lung, testis and ovaries. Peripheral blood counts and blood chemistries (including glucose, Bun, ALT, and Total Protein) were within normal limits. Glucose levels were 245 ± 24 mg/dl for knock-out mice and 233 ± 50 mg/dl for wild type mice.

### Proliferative behavior of MEFS

**Figure 4:**
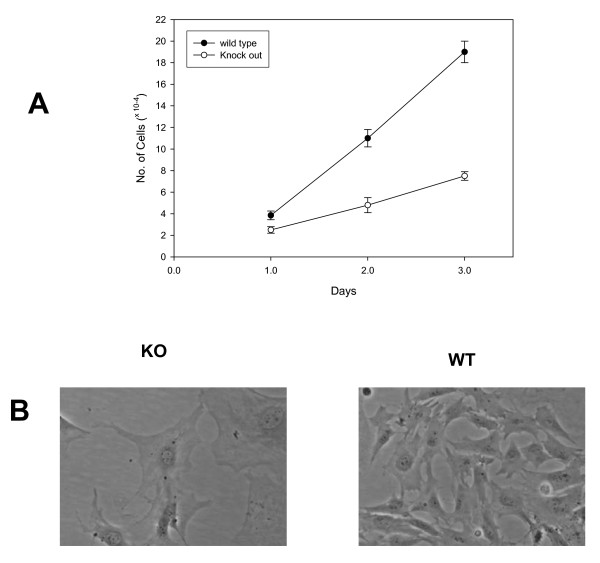
**Reduced cellular proliferation of MEFs from wild type and Ebp1 knock out mice**. A. MEFs were plated as described in the Materials and Methods and counted for 3 consecutive days. B. Morphological appearance of Ebp1 knock out (KO) and wild type (WT) MEFs after three passages.

As Ebp1^-/- ^mice were small and the *Ebp1(pa2g4) *gene was originally identified as a proliferation associated gene, we examined the proliferative behavior of 12.5 day mouse embryo fibroblasts. Under the standard culture conditions used, the number of MEFs from *Ebp1*^-/- ^mice was lower at all the time points tested (Fig. 4A). In addition, we were able to propagate wild type MEFs for 5 passages, but *Ebp1*^-/- ^MEFs for only 3. A senescent morphology was observed at passage 3 for knock out MEFs (Fig. 4B).

### Target gene and global expression profiling in MEFs

**Figure 5:**
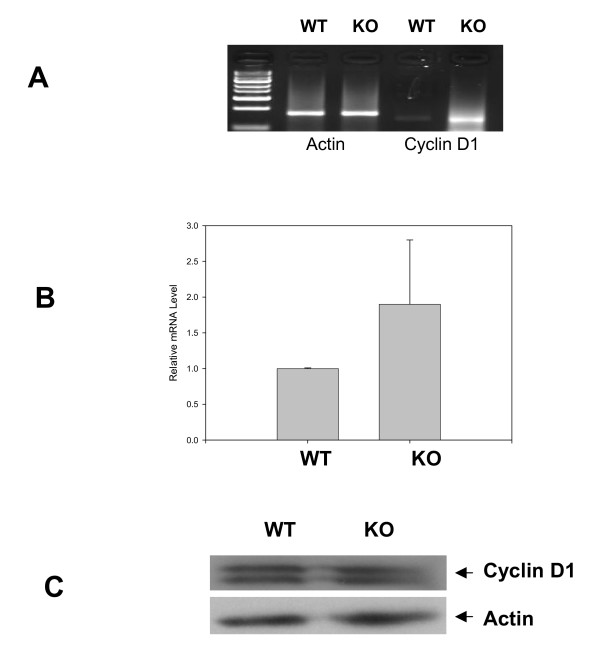
**Validation of differential Cyclin D1 expression in wild type and Ebp1 knock out MEFs**. A. Conventional PCR analysis of Cyclin D1 and actin mRNA in wild type and knock out MEFs. B. Real Time quantitative RT-PCR analysis of Cyclin D1 mRNA. The relative level of Cyclin D1 was normalized to β actin. Results are representative of 2 experiments using different sets of cells. C. Expression of Cyclin D1 protein in wild type and knock out MEFs. Lysates of *Ebp1 *knock out or wild type MEFs were resolved by SDS-PAGE and analyzed by western blotting with the indicated antibodies.

To obtain additional information about the cellular pathways involved in the phenotype of the MEFs, we performed global expression profiling of matched 12.5 day MEFs. The expression data have been deposited with the GEO data base  as GSE13718. A total of 99 genes were changed more than two fold (500 expression units minimum). Among the transcripts involved in growth regulation, we found that several members of the insulin, EGFR, Cyclin and MAPK families were significantly changed (Table 1). To verify these findings, we first examined the expression of Cyclin D1 which has previously been shown to be repressed by ectopic expression of Ebp1 in human breast cancer cell lines [24,8]. We found by RT-PCR and quantitative PCR analysis that Cyclin D1 mRNA was increased (Fig. 5A, B). However, Cyclin D1 protein levels were equal in wild type and knock out MEFs reflecting the complex regulation of Cyclin D1 protein expression [25] (Fig. 5C).

**Table 1:** Expression of proliferation associated genes on Ebp1 knock out versus wild type MEFs

**Accession Number**	**Gene Name**	**Fold Change**	**P**
Insulin Family			

BB787243	insulin-like growth factor binding protein 4	-2	.0014
BG075165	insulin-like growth factor	-2	.0002
AV175389	insulin-like growth factor binding protein 3	-2.3	.0002
MAPK Signaling			
			
NM_016719	Grb14	+1.9	.0002
NM_009231	Son of sevenless homolog 1	+5.1	.0007
X58876.1	mdm2 protein	+2.5	.0004
AF022072	Grb-10	+2.2	.003
Cyclin Family			
			
NM_007631	cyclin D1	+2.1	.0007
NM_009877	cyclin-dependent kinase inhibitor 2A (Cdkn2a),	+3.2	.0004
Growth Factors and Growth Factor Receptors			
			
L07264	heparin-binding EGF-like growth	+2.2	.0001
NM_007540	brain derived neurotrophic factor (BDNF)	+3.0	.0002
AW537708	PDGF receptor, alpha polypeptide	-3.2	.003

Three regulators of the insulin signaling pathway were also found to be significantly decreased. These include IGF-1, IGFBP-3 and IGFBP-4. The levels of IGF-1 were measured by ELISA in conditioned media derived from wild type and knock out MEFs. The concentration of IGF-1 in conditioned media from 3 day cultures of MEFs was 305 ± 25 pg/ml for knock out cells and 480 ± 32 pg/ml for wild type MEFs.

### Alterations of EGF-regulated signaling pathways

**Figure 6:**
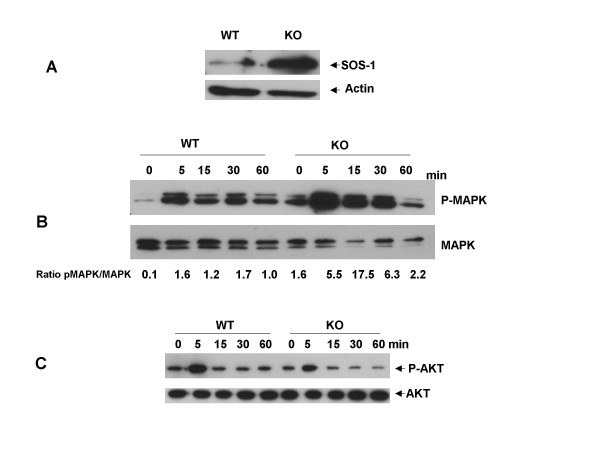
**Activation of kinase cascades in Ebp1 wild type and knock out MEFs**. A. The expression of SOS-1 in logarithmically growing wild type and knock out MEFs was measured by Western blot analysis for SOS-1 and actin as indicated. B. WT and KO MEFs were serum starved for 24 hours and then stimulated with EGF (20 ng/mL) for the indicated times. Cells were lysed and Western blot analysis performed to examine the activation states of MAPK (B) and AKT (C) using phospho specific antibodies directed against each protein. The expression of total MAPK and AKT was also measured as indicated. MAPK blots were analyzed by densitometry using Image-J software. Results presented in Panel B underneath each lane are the ratios of pixel densities of the pMAPK to MAPK bands.

Our microarray data indicated that two members of the MAPK signal transduction pathway, SOS1 and Grb14 were upregulated in knock-out as compared with wild type MEFs. We verified by Western blot analysis that SOS was increased in Ebp1 knock out MEFs as compared with wild type cells (Fig. 6A). We therefore tested if the cellular response to EGF would be changed in Ebp1 knock out MEFs. Murine MEFs are known to express EGFR and EGFR mRNA expression was not changed in Ebp1 knock outs (data not shown)[26]. Cells were treated with EGF (20 ng/ml) for the times indicated and MAPK activation assessed by detection of phosphorylated MAPK by Western blot analysis. The results indicated that both wild type and knock out MEFs could respond to EGF stimulation as evidenced by phosphorylation of MAPK. However, MAPK was basally phosphorylated only in knock out MEFS (Fig. 6B). EGF-induced AKT phosphorylation in wild type and knock out cells was also determined using a phospho specific AKT antibody. The response to EGF was equivalent in wild type and knock out cells (Fig. 6C).

### Changes in expression of proliferation associated genes in adult target tissues

**Figure 7:**
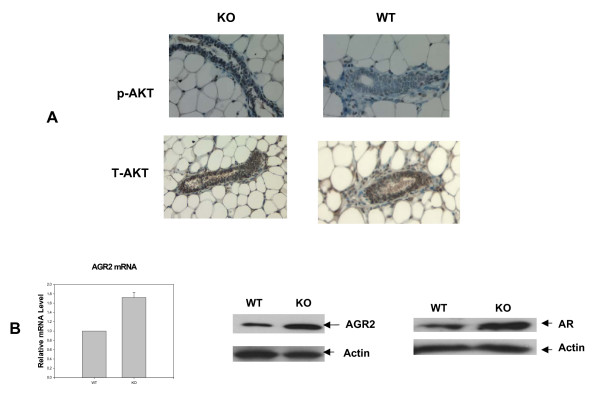
**Changes in expression of Ebp1 target genes in adult tissue**. A. Number 4 mammary glands were isolated from 10 week old wild type (WT) or Ebp1 knock out (KO) mice. Immunohistochemical analysis was performed for phospho AKT (p-AKT) or total AKT (T-AKT) as indicated. B. Expression of AR and AR regulated genes in prostates. The expression of AGR2 in the anterior prostates of age matched wild type and Ebp1 knock out mice were measured by real-time PCR (left panel) or Western blot (middle panel) analysis. AR levels were measured in lysates of anterior prostates by Western blot analysis (Right panel).

Previous *in vitro *work with Ebp1 has used breast and prostate cancer cell lines derived from adults. In these tissues, the expression of Cyclin D1[24], AGR2 [27] and androgen receptor [14] have been shown to be regulated by Ebp1. We therefore examined expression of these genes in adult mammary and prostate epithelium. We found that in mammary epithelial cells, Cyclin D1 protein levels were not changed in knock out mice as compared to wild type (data not shown). However, the phosphorylation of AKT was greatly increased in Ebp1^-/- ^mammary tissue (Fig. 7A). In prostate cancer cell lines, the expression of the androgen receptor and the metastasis associated protein AGR2 has been demonstrated to be decreased by ectopic expression of Ebp1. We found that AGR2 mRNA (Fig. 7B, right panel) and protein (Fig. 7B, middle panel) expression and AR protein expression (Fig. 7B, right panel) were increased in prostates of Ebp1 knock out mice as compared to age matched controls.

**Figure 8:**
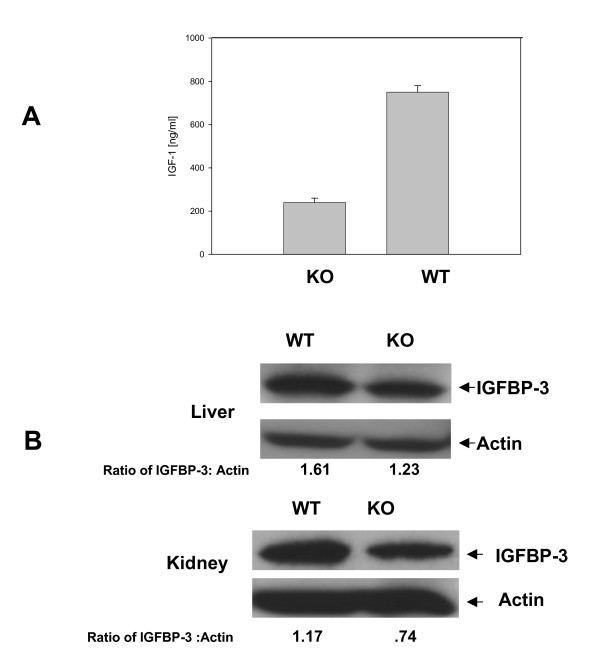
**Alterations of insulin signaling pathways in adult Ebp1 knock out mice**. A. Serum IGF-1 as measured by ELISA. IGF-1 levels in serum from Ebp1 knock out and wild type mice were assessed by ELISA as described in the Materials and Methods. B. Liver and kidneys of 3 month old wild type (WT) and knock out (KO) mice were analyzed by Western blotting for expression of IGFBP-3 or actin as indicated. Blots were analyzed by densitometry using Image-J software. Results presented underneath each lane are the ratios of pixel densities of IGFBP-3 to actin bands.

Finally, based on data from MEFs, we examined serum levels of IGF-1 from age matched wild type and knock out mice by ELISA assays. IGF-1 was reduced to 66% of wild type levels in serum of 6 month old mice (Fig. 8A). In addition, Western blot analysis of adult liver and kidney (3 month old mice) indicated that IGFBP-3 was reduced in knock-out versus wild type organs (Fig. 8B).

## Discussion

To characterize the physiological role of Ebp1, we generated Ebp1 deficient mice exhibiting a gene trap introduced into intron 2 of the *Ebp1 *gene. The gene trap leads to deletion of all but the first 20 amino acids of Ebp1 which have no biological function on their own. The most striking feature of the Ebp1^-/- ^phenotype was the small size of the mice. It is of interest to note that inhibition of expression of the potato homologue of Ebp1 also results in a dwarf phenotype [4]. *Ebp1*^-/- ^pups were on average 25% smaller than their wild type and heterozygous littermates until Day 30, the approximate onset of puberty. Mice were well proportioned with no skeletal abnormalities other than a kinky tail in approximately 1/3 of the mice. An unusual feature of our *in vivo *analysis is the fact that the reduced weight was recovered after the prepubescent stage, while Ebp1 is still normally transcribed and translated and therefore likely to have a regulatory role. Although we are unable to explain the mechanism underlying this recovery, we report this observation as a possible example of temporal restricted competence in growth regulation of specific cell types by Ebp1. This phenotype is similar to that observed for the OTX 1 gene, a homeobox containing gene that *in vitro *activates transcription of GH, FSH, and LH [28].

It is of interest that the phenotype of Ebp1 knock out animals also resembles that of the *IMP1 *knock out mice [29]. IMP1 belongs to a family of RNA binding proteins implicated in mRNA localization, turnover, and translational control. These mice also exhibit a dwarf phenotype with reduced proliferation of knock out MEFs. In these mice, growth retardation is related to a hypoplastic small intestine. In contrast, Ebp1 mice exhibit a transient dwarfism and their intestines are both grossly and histologically normal at 10 weeks. However, we were unable to assess intestinal anatomy and histology at earlier time points, and it remains possible that abnormalities occur when the weight differences are most marked. Ebp1 has also been shown to be an RNA binding protein with a sigma- 70 like motif. This motif has been identified as a new RNA binding domain unique to the superfamily of Imp4 or Brix proteins that are required for ribosome biogenesis [30]. A role for Ebp1 in ribosomal biogenesis is supported by studies indicating its localization in the nucleolus, its association with 5 S ribosomal RNA, and its complexing with proteins such as B23 which are involved in ribosome processing[19]. Whereas the significance of RNA binding proteins in spatial and temporal control of gene expression during development is evident in invertebrate organisms, the function of RNA binding proteins during mammalian development is less clear. With the exception of FMRP, Dazla, and Nova-1, which are associated with mental retardation, azospermia, and neuronal viability respectively [31-33], the majority of the mammalian RNA binding proteins are constitutively expressed and exhibit a relative broad RNA-binding specificity. Further work is needed to uncover the role that Ebp1's RNA binding activity has in the development of this phenotype and the target RNAs that specifically interact with Ebp1.

The molecular pathways leading to dwarfism are likely to involve several mechanisms. As reduced growth was manifested in the MEFs from Day 12.5 embryos, we used these cells to measure changes in gene expression associated with loss of Ebp1. Global gene expression analysis indicated that expression of IGF-1 and the IGF binding proteins IGFBP3 and 4 was decreased. The *in vivo *growth promoting role of the IGFs has been demonstrated conclusively from the dwarfing phenotypes observed after targeted mutagenesis of the mouse *Igf1 *and *Igf1r *genes. [34]. IGF-1 functions both during embryonic and post natal development. IGF-1 mutants exhibit a birth weight that is 60% of normal and the difference between their size and that of wild type mice increases progressively with age [35]. The Ebp1 phenotype is not as severe as that observed in IGF-1 deficient mice. The function of IGFBPs in cell growth is more complex. However, one role is to sequester and stabilize IGF-1 [36]. Thus, decreases in IGFBPs may result in destabilization of IGF-1 and may be partially responsible for lower levels of serum IGF-1.

We found that MAPK was constitutively phosphorylated in MEFs derived from knock out mice. This constitutive phosphorylation was associated with the overexpression of SOS-1, an upstream activator of the Ras -MAPK signal transduction pathway. It is possible that overexpression of SOS-1 led to increased basal phosphorylation of MAPK. In contrast, both the basal and HRG-induced activation of the MAPK pathway is increased in *EBP1 *transfected breast cancer cell lines [11]. The reasons for these apparently discordant findings may be related to the ligand used, the differential expression of ErbB receptors in breast cancer cells versus fibroblasts, and the cellular background.

We have previously shown that inhibition of Ebp1 expression results in increases in phospho AKT in human breast cancer cell lines, whereas ectopic expression of *ebp1 *inhibits HRG induced AKT activation [15]. Here, we found that Ebp1 had no effect on AKT phosphorylation after EGF stimulation of MEFs. However, AKT was constitutively phosphorylated in mammary epithelial cells of knock-out mice. The mechanism of the increase in AKT phosphorylation is unclear. Paradoxically, Ahn et al [16] have shown the binding of Ebp1 to nuclear AKT increases AKT phosphorylation [37] in PC-12 neuronal cells. It is not known if Ebp1 binds AKT in normal mammary epithelial cells or affects its phosphorylation. These studies point up the importance of tissue context in the activation of specific signaling molecules. As activation of AKT is important in development of mammary adenocarcinoma [38], we are currently monitoring breast tumor incidence with age in Ebp1 knock out mice. We have not yet seen increased tumor incidence in a small cohort of mice that have been carried until one year.

Finally, our laboratory has demonstrated that ectopic expression of *Ebp1 *results in decreased expression of AR and AR target genes and inhibition of growth of prostate cancer cells both *in vivo *and *in vitro *[14]. In the current study, deletion of the *Ebp1 *gene led to increased expression of AR in prostates of adult mice and upregulation of the metastasis associated gene *AGR2*. AR plays a key role in the development and progression of prostate cancer, and we anticipate that the *Ebp1 *knockout mouse may have a hyperactivated AR signaling axis that may result in increased incidence of prostate cancer. Also with regard to prostate cancer, decreased expression of IGFBP-3 is correlated with increased risk of developing prostate cancer[39]. Western blot analysis verified that *Ebp1 *knockout led to reduced protein levels of IGFBP-3 in liver and kidney. We were unable to detect IGFBP-3 in prostates of either wild type or knock out mice, but studies are ongoing to assess if there are changes in IGFBP-3 expression in the prostates of knock out mice. We are also monitoring the incidence of prostate cancer of the Ebp1^-/- ^mice as they age. Nevertheless, current studies, together with our previous reports, strongly suggest that Ebp1, as an endogenous regulator of ErbB-AR crosstalk, may regulate expression of a series of genes that are involved in aggressive prostate cancer growth.

## Conclusion

In summary, although Ebp1 appears to be dispensable for prenatal development, loss of Ebp1 affects post-natal growth. This growth delay may be related to changes in IGF-1 and IGFBP levels and to changes in key components of cellular proliferation pathways. Of interest, two genes involved in prostate cancer progression, androgen receptor and AGR2, are increased in prostates of Ebp1 knock out mice. Our Ebp1^-/- ^mouse line represents a new *in vivo *model to investigate Ebp1 function in the entire organism.

## Methods

### Gene trap insertion in ES cells and generation of Ebp1^-/- ^mice

Embryonic stem (ES) cells (129/SvEvBrd) with a retroviral gene trap vector insert (VICTR20) into the Ebp1 locus (Omnibank no. OST 186047) were generated as described previously [23] (Lexicon Genetics, Woodlands TX). The location of the gene trap insertion was determined by sequence analysis. The *Ebp1 *targeted ES cells were microinjected into C57BL/6 albino blastocysts, followed by transfer to a foster mother (C57Bl/6) to generate chimeric animals. Chimeric males were mated with C57Bl/6 albino females, and F1 agouti pups were analyzed for the presence of the transgene by PCR analysis of 100 ng of tail genomic DNA with the following primers to detect mutant and wild type Ebp1 : For the mutant gene primers directed against viral LTR2 (Forward):AAATGGCGTTAAGCTAGCTTGC: and Reverse 5'CCTTTAATCCCAGCACTCTGGAACG3'. For wild type Ebp1 Forward PCR5'TAGAGCCTCTACAGTGTTTTGAGG3' and Reverse 5'CCTTTAATCCCAGCACTCTGGAACG3'. For approximate positions of primers see Fig 1. PCR amplification of wild type Ebp1 sequences leads to a 308 bp product. Amplification of LTR2 and Ebp1 lower leads to amplification of a 253 bp product to detect the insert LTR2. Heterozygous *Ebp1*^+/- ^mice were crossed to obtain homozygous *Ebp1*^-/- ^mice. The strain was maintained on a 50% C57BL/6–50% 129SvEvBrd background. The mice were bred on a 10 hour light:14 hour dark cycle and had free access to drinking water and standard chow. All mice were generated and maintained in accordance with institutional guidelines approved by the University of Maryland Baltimore Animal Care and Use Committee.

### Isolation of Mouse Embryo Fibroblasts (MEFS) and proliferation assays

Embryos (12.5 days)were minced and incubated in 0.25% trypsin and 5 U of DNAse I for 37°C for 30 min. Dulbecco's MEM supplemented with 10% fetal bovine serum and penicillin and streptomycin were added to the cell suspension and cells centrifuged at 1000 rpm for 10 min. The pellet was resuspended in medium and cells were grown in MEM and passaged by trypsinization. Cell proliferation was measured by plating MEFs from each genotype at a density of 2 × 10^4 ^cells per 35 mm dish. Viable cells were counted by Trypan blue exclusion using a hemocytometer. Cells were used in passage 2 and 3. For EGF stimulation experiments, passage 2 MEFs were plated in 6 well plates in complete media. Cells were serum-starved overnight and then stimulated for the indicated times with EGF (Sigma, St. Louis, MO) at 20 ng/ml.

### Microarray Analysis

First and second strand cDNA were synthesized from 5–15 μg of total RNA at Genome Explorations (Nashville, TN) using the SuperScript Double-Stranded cDNA Synthesis Kit (Gibco Life Technologies) and an oligo-dT_24_-T7 (5'-GGC CAG TGA ATT GTA ATA CGA CTC ACT ATA GGG AGG CGG-3') primer according to the manufacturer's instructions. cRNA was synthesized labeled with biotinylated UTP and CTP by in vitro transcription using the T7 promoter coupled double stranded cDNA as template and the T7 RNA Transcript Labeling Kit (ENZO Diagnostics Inc.). The fragmented cRNA was hybridized to U133A oligonucleotide arrays (Affymetrix) containing ~33,000 full length annotated genes together with additional probe sets designed to represent EST sequences. The arrays were then stained with phycoerythrein conjugated streptavidin (Molecular Probes) and the fluorescence intensities were determined using a laser confocal scanner (Hewlett-Packard). The scanned images were analyzed using Microarray software (Affymetrix). Sample loading and variations in staining were standardized by scaling the average of the fluorescent intensities of all genes on an array to constant target intensity for all arrays used. The signal intensity for each gene was calculated as the average intensity difference, represented by [E(PM - MM)/(number of probe pairs)], where PM and MM denote perfect-match and mismatch probes. Data Analysis was conducted using Microarray Suite 5.0 (Affymetrix) following user guidelines. Only genes with a mimmum expression level of 500 were included in this analysis. Genes whose expression varied more than two fold with a p value of < 0.05 were considered to be significantly different between the two cell lines.

### Real Time Quantitative Reverse-Transcription and Conventional PCR Analysis

The method of Nakanishi et al [40]was used as previously described. Real-time quantitative RT-PCR was performed on the LightCycler (Roche) platform to determine relative mRNA levels of genes under study. The following forward and reverse primers were selected using Primer Express software and synthesized by the Core Laboratory of University of Maryland School of Medicine: Ebp1, sense: 5'-GCACGCCAATAGAAGG-3'and antisense: 5'-GTAAACGGCATGGCATC-3', β-Actin, sense: 5' GCT ATC CAG GCT GTG CTA TC-3' and antisense TGT CAC GCA CGA TTT CC-3', Cyclin D1, sense 5'-GCAGCACCCGGTCGTTGAGGA-3' and antisense:5'-TCCGGAGACCGGCAGTACA-3', AGR2, sense: 5' ATTGGCAGAGCAGTTTGTCC and antisense: 5'GAGCTGTATCTGCAGGTTCGT[41]. A SYBR Green PCR Kit was used (Applied Biosystems, Foster City, CA) and the analyses were performed in duplicate or triplicate in a total volume of 15 μl including 0.9 μl of 25 mM MgCl_2_, 1.5 μl SYBR Green I, 0.3 μl Enzyme Mix, 0.75 μl of each primer (50 ng/μl) and 2 μl of cDNA synthesized with random hexamers. Target mRNA values were normalized using β-actin mRNA as an internal control. The relative quantitation of gene expression was performed using the comparative ΔΔC_t _(threshold method) using β-actin as an internal control [42].

Conventional PCR analysis was performed as previously described [2]. PCR products were visualized on 1.2% agarose gels stained with ethidium bromide.

### Western Blot Analysis

Briefly, total cell extracts were prepared by direct lysis of cells with buffer containing 50 mM Tris-HCl (pH 7.4), 1 mM EDTA, 250 mM NaCl, 1% Triton X-100, 0.5 mM DTT and 1 mM PMSF. Proteins concentrations were measured using a detergent compatible kit (BioRad, Hercules, CA). Proteins were resolved by SDS-PAGE and analyzed by Western blotting as described [6]. The Ebp1 antibody was from Upstate (Lake Placid, NY), Cyclin D1, IGFBP-3, SOS-1 and Androgen Receptor antibodies from Santa Cruz (Santa Cruz, CA), the phospho AKT and AKT antibodies from Cell Signaling (Beverly, MA), the phospho MAPK and MAPK from Promega (Madison, WI), the AGR2 antibody from Dr. Charles Young, Mayo Clinic[43], and the polyclonal antibody to actin from Sigma (St. Louis, MO). Images were quantified using IMAGE-J software (NIH).

### Measurement of IGF-1 levels

IGF-1 levels in serum and conditioned media were determined using an IGF-1 ELISA kit from R&D Systems (Mpls, MN) as directed by the manufacturer. For conditioned media preparation, passage 2 MEFs were plated at a density of 2 × 10^4 ^cells per 35 mm dish in 3 ml of complete media. Media were collected 3 days later.

### Immunohistochemical staining

Mammary glands (#4) were excised and fixed in 10% buffered neutral formalin. Sections of formalin-fixed, paraffin-embedded tissues were cut to 5 μm. Slides were stained with Cyclin D1, phospho AKT and total AKT antibodies [43]diluted 1:100 using the standard avidin-biotin method (Vecta-Stain Kit, Vector Labs, Burlinghame, CA) with Harris hematoxylin as a counterstain.

### Statistical Analysis

Data were analyzed using a two-tailed Students t-test and a p < 0.05 was deemed statistically significant.

## Authors' contributions

YZ conceived the study, carried out animal breeding and weight and PCR analysis, performed Western blot and microarray assays and edited the manuscript. YL carried out PCR analysis, immunohistochemical and western blot analysis. HZ collection and assembly of data; HZ performed Western blot analysis, qRT-PCR analysis and participated in the microarray studies. M-HL participated in design of the study and development of PCR analysis for detection of the gene trap insertion. ZL participated in the design of the study and performed the statistical analysis. BH conceived the study, participated in its design and coordination, and helped draft the manuscript. AWH conceived the study, carried out proliferation and ELISA assays, participated in mouse breeding and weighing and drafted the manuscript. All authors read and approved the final manuscript.
